# How women living with HIV in the UK manage infant-feeding decisions and vertical transmission risk – a qualitative study

**DOI:** 10.1186/s12889-024-19581-9

**Published:** 2024-08-06

**Authors:** Bakita Kasadha, Lisa Hinton, Shema Tariq, Farai Nyatsanza, Angelina Namiba, Nell Freeman-Romilly, Tanvi Rai

**Affiliations:** 1 Nuffield Department of Primary Care Health Sciences, University of Oxford, Oxford, UK; 2 Institute for Global Health, University College London (UCL), London, UK; 3 Central and North West London NHS Foundation Trust, London, UK; 4 Cambridgeshire Community Services NHS Trust, Cambridge, UK; 5 4M Mentor Mothers Network, London, UK; 6 Oxford University Hospitals NHS Foundation Trust, Oxford, UK

**Keywords:** HIV prevention, Vertical transmission, Infant-feeding, Breastfeeding, Mother-to-child transmission, HIV

## Abstract

**Background:**

The World Health Organization (WHO) recommends that women with HIV breastfeed for a minimum of one year. In contrast, across high-income countries, HIV and infant-feeding guidelines recommend exclusive formula feeding if parents want to avoid all risk of postpartum transmission. However, recently these guidelines (including in the United Kingdom (UK)) increasingly state that individuals with HIV should be supported to breast/chest feed if they meet certain criteria; such as an undetectable maternal HIV viral load and consent to additional clinical monitoring. Between 600 and 800 pregnancies are reported annually in women with HIV in the UK, with low rates of vertical transmission (0.22%). Informed infant-feeding decision-making requires clinical support. Currently, little research addresses how individuals with HIV in high-income countries navigate infant-feeding decisions with their clinical teams and familial and social networks, and the resources needed to reach an informed decision.

**Methods:**

Semi-structured remote interviews were conducted between April 2021 – January 2022 with UK-based individuals with a confirmed HIV diagnosis who were pregnant or one-year postpartum. Using purposive sampling, pregnant and postpartum participants were recruited through NHS HIV clinics, community-based organisations and snowballing. Data were analysed thematically and organised using NVivo 12.

**Results:**

Of the 36 cisgender women interviewed, 28 were postpartum. The majority were of Black African descent (*n* = 22) and born outside the UK. The majority of postpartum women had chosen to formula feed. Women’s decision-making regarding infant-feeding was determined by (1) information and support; (2) practicalities of implementing medical guidance; (3) social implications of infant-feeding decisions.

**Conclusion:**

The evolution of UK HIV and infant-feeding guidelines are not reflected in the experiences of women living with HIV. Clinicians’ emphasis on reducing the risk of vertical transmission, without adequately considering personal, social and financial concerns, prevents women from making fully informed infant-feeding decisions. For some, seeking advice beyond their immediate clinical team was key to feeling empowered in their decision. The significant informational and support need among women with HIV around their infant-feeding options must be addressed. Furthermore, training for and communication by healthcare professionals supporting women with HIV is essential if women are to make fully informed decisions.

**Supplementary Information:**

The online version contains supplementary material available at 10.1186/s12889-024-19581-9.

## Background

Significant advances in HIV treatment have rendered HIV a chronic and manageable disease, with life expectancy similar to those without HIV [1]. Data show that when on effective antiretroviral therapy (ART), people cannot transmit HIV via condomless sex [2]. While antenatal HIV testing and maternal ART have led to a substantial and sustained decrease in infants acquiring HIV perinatally [3] by preventing the transmission of HIV during pregnancy and childbirth (when the viral load is undetectable) [4], current data show that the transmission risk through breastfeeding is not zero. PROMISE, a multicentre randomised controlled trial in African countries and India, reported a 0.3% and 0.6% risk of HIV transmission when mothers on ART breastfeed at six months and 12 months respectively [5]. Although there is a paucity of evidence about mothers who breastfeed in high-income settings [6]; limited data in North America and the UK report no cases of vertical transmissions among breastfeeding mothers with an undetectable viral load [7–9].

In 2016, the World Health Organization (WHO) and United Nations Children’s Fund (UNICEF) produced guidelines recommending that mothers living with HIV breastfeed for at least 12 months [10]:

*‘Mothers living with HIV should breastfeed for at least 12 months and may continue breastfeeding for up to 24 months or longer (similar to the general population) while being fully supported for ART adherence (see the WHO consolidated guidelines on ARV drugs for interventions to optimize adherence).’ (2016; p3)*

These guidelines are primarily intended for low income and high HIV prevalence settings, where the potential risk of postpartum transmission of HIV via breastmilk is understood to be lower than the risk of infant malnutrition or death because of limited access to clean water. In contrast, across several high-income countries, where access to safe drinking water is assumed, mothers living with HIV have been advised to formula feed their babies exclusively [6, 11–15]. While the risk of HIV transmission is low, there are significant benefits associated with breastfeeding. In its 2023 Breastfeeding Series, The Lancet stated that ‘Human infants and young children are most likely to survive, grow and develop to their full potential when breastfed’ [16]. The data show that breastfed babies have stronger immune systems and reduced risk of developing certain chronic health conditions in the long-term (than their formula fed counterparts) [17]. Evidence also suggests that breastfeeding has a small positive effect on a child’s intelligence [18]. Breastfeeding lowers the risk of post-partum depression and promotes maternal wellbeing more broadly [19, 20]. However, those who experience breastfeeding difficulties are more likely to experience post-partum depression and bonding difficulties in the first six months post-partum [21, 22] and long-term breastfeeding is associated with ‘prolonged earning losses’ [23, 24]. For the general population, combination or mixed feeding is viewed as beneficial in finding a balance between the burdens of either exclusive formula feeding or exclusive breastfeeding [25].

At the time of data collection, the British HIV Association (BHIVA), the organisation that sets the UK HIV treatment guidelines, stated that individuals should be encouraged to formula feed, to remove all risk of HIV transmission however ‘…women who are virologically suppressed on ART with good adherence and who choose to breastfeed should be supported to do so, but should be informed about the low risk of transmission of HIV through breastfeeding in this situation and the requirement for extra maternal and infant clinical monitoring (2020; p. 95). These guidelines, which have been in effect since 2018, advise breastfeeding cessation by six months.

Between 600 and 800 pregnancies occur in women with HIV annually in the UK (where ART is free to access to all people regardless of immigration status), with low rates of vertical transmission (0.22%) [9, 26, 27]. The majority of pregnant women are aware of their status and are on ART at the time of conception; 65% are Black African migrants, mostly from countries with high HIV population prevalence where breastfeeding (regardless of HIV status) is advised [9]. Due to the low risk of vertical transmission and 2018 guideline change, women living with HIV in the UK are increasingly considering breastfeeding [9, 27, 28]. A survey found that 38% would like to breastfeed and 66% felt forced to invent a reason why they were not breastfeeding [29].

Although the proportion of women living with HIV who breastfeed is low at 3.1%, it is likely to increase [29, 30]. Our study (NOURISH-UK) is the first to investigate infant-feeding decision-making among women living with HIV, since the updated guidelines were published. This paper contributes to addressing this data gap, alongside our other findings (published elsewhere) which shows how the role of fathers and partners is underestimated in the decision-making process [31] and that both formula feeding and breastfeeding can be seen as transgressive practices within different contexts, placing complex burdens on women deciding between these options [32]. In this paper we explore how women’s infant-feeding decisions are shaped by their own understanding and perceptions, and through the interactions with their healthcare professionals (within HIV care, paediatrics, maternity wards etc.).

## Methods

NOURISH-UK was an in-depth qualitative study comprising semi-structured interviews with women living with HIV in the UK. BK and TR collected the data and made field notes during and immediately following each interview. Both are women from racially minoritised backgrounds and are non-clinical researchers. They have extensive experience in conducting qualitative research and have worked in HIV research over several years.

### Recruitment

Participants were recruited from across the UK via pre-authorised Participant Identification Centres (PIC) at National Health Services (NHS) Trusts providing HIV specialist care, as well as through HIV charities, snowballing and personal contacts. We distributed participant recruitment packs to the PICs, and a flyer with information about the study via the team’s existing professional networks. BK also attended online mother and baby groups facilitated by HIV charities, to further promote the study and increase recruitment.

Inclusion criteria were (a) an HIV diagnosis, (b) age ≥ 18 years (c) currently pregnant or up to one-year postpartum and (d) currently living in the UK. All individuals who met these inclusion criteria and were interested in study participation and gave fully informed consent were included. Recruitment continued until data saturation was reached [33].

Although we sought to be transgender inclusive, we did not hear from transgender, non-binary or gender diverse parents. We therefore use ‘mother’ and ‘pregnant women’ when discussing our data, while recognising that birthing parents living with HIV of other genders may share some similar experiences.

### Interviews

We conducted semi-structured interviews between April 2021 and January 2022. Due to the COVID-19 pandemic, all interviews were remote. Each participant chose whether they preferred a meeting via telephone or MS Teams (approximately half chose the former and half the latter). Telephone interviews were offered to avoid exclusion (due to digital inequalities) and for a level of privacy for participants who did not want to show their face. Each participant had one interview with one of the interviewers. The majority of participants were alone at the time of their interview, however some were accompanied by their infants and a minority had their partners present. BK and TR conducted the interviews. Each interview lasted approximately an hour and were digitally recorded. We took fully informed (both verbal and written) consent from all participants and they received a £20 gift voucher for their participation. Phone calls were made by the interviewers (to ensure participants did not use their minutes), and participants facing digital exclusion were offered digital devices (to be couriered to their home) to enable their participation.

The topic guide covered the following broad areas: participant background (relationship and family status, learning about HIV diagnosis); experiences of most recent pregnancy, and previous pregnancies (as appropriate); knowledge about latest HIV and infant-feeding information; conversations with healthcare professionals and within personal support networks regarding infant-feeding; and infant-feeding decision-making and experiences, and strategies for support.

### Analysis

Verbatim transcripts were shared with participants for accuracy. BK and TR analysed the data thematically [34], incorporating a mind-mapping approach known as the one sheet of paper (OSOP) method [35] to support critical, reflective analysis. Both inductive and deductive thematic analyses were used to develop a coding framework which was applied iteratively to the data. BK and TR grouped related extracts from transcripts around developing themes, which were then analysed further using mind-maps that they developed independently of each other. These were then discussed to resolve any differences. The OSOP mind mapping method enables all relevant data to be included in the thematic analysis, and is a thorough and auditable approach [35]. The themes from our analysis were further validated through multiple discussions with our a Patient and Public Involvement (PPI) Group, composed of five mothers living with HIV, and a multi-stakeholder advisory panel of over 20 professionals including HIV specialists, HIV support groups and non-HIV medical professionals. Details about our stakeholder engagement are reported elsewhere [36].

Data files were managed using NVivo 12. Throughout this paper, we present illustrative quotes, with pseudonyms chosen by participants themselves.

## Results

### Participant characteristics and population

Of the 45 individuals who provided verbal consent to be interviewed, 36 cisgender women provided post-interview written consent to be included in this study (see Table 1); eight were pregnant and 28 postpartum. The majority of postpartum women had an undetectable HIV viral load at the time of birth (*n* = 26). Aside from five participants who had been diagnosed with HIV in their most recent pregnancy, the rest had received their HIV diagnosis before the changed 2018 BHIVA infant-feeding guidelines (which, as detailed above, support breastfeeding under certain circumstances).

**Table 1:** Participant characteristics

Characteristics (self-described)	Participants (*n* = 36)
**Age (years)**	
18–24	2
25–29	6
30–34	8
35–39	10
40–44	10
**Ethnicity**	
Asian	3
Black African	22
Black Caribbean	2
White British	6
White other	2
Not known / stated	1
**Region of birth**	
Africa	21
Mainland Europe	2
UK and Ireland	11
Elsewhere	2
**Diagnosed during most recent pregnancy**	
Yes	5
No	31
Timing of HIV diagnosis*	
< 1 year	2
1–10 years	22
11 to 25 years	9
26 + years^a^	3
Afford basic needs*	
All / most the time	16
Some/ none of the time	16

*missing data due to non-response

^a^Diagnosed before the availability of modern, highly-effective antiretroviral therapies (ART)

The majority (*n* = 28) of participants were postpartum and had chosen to formula feed. They were mainly motivated by removing all risk of HIV transmission (especially the two women who had not achieved virological suppression at the time of birth). A few also expressed that formula feeding enabled them to share feeding duties with their partner and free formula alleviated financial concerns. However, not all women were able to access free formula via their clinics or local HIV charities, which presented a significant financial challenge for some wishing to formula feed.

The minority of postpartum participants who had wanted to breastfeed (*n* = 8) were mainly motivated by the health benefits of breastmilk to their baby (and one to herself and baby, discussed later). Those who had successfully breastfed also spoke of the bonding they experienced with their infant through breastfeeding, as well as breastfeeding allowing them to conform to personal, societal and cultural expectations of motherhood. However, a few women stopped breastfeeding earlier than planned due to a combination of a lack of tailored lactation support, unclear guidance, issues with breastmilk supply and/or cracked nipples (discussed later).

In the following sections, we present our participants’ experiences of their infant-feeding decision-making, particularly with regards to managing HIV transmission risk. We focus on how their decisions and experiences were shaped by their understanding of risk and current BHIVA guidelines, communication with healthcare professionals, friends and family, and their personal circumstances. Our findings reveal how decision-making was a dynamic process, rather than situated in one point in time. Decisions were often revisited and re-interrogated at different stages of pregnancy, childbirth and postpartum.

We now focus on three themes: (1) information and support; (2) practicalities of implementing medical guidance; (3) social implications of infant-feeding decisions (Table 2).

**Table 2:** Common thematic subjects and sub-themes

Common thematic subjects	Sub-themes
Information and support	Healthcare professionals sharing information about the BHIVA guidelines and the feeding options available
	Women’s knowledge of and perception of the guidelines, transmission risk and research
	Making the ‘healthiest’ choice for the baby
Practicalities of implementing medical guidance	Breastfeeding ‘safely’
	Congruence between infant-feeding decision and infant-feeding experiences
	Incongruence between infant-feeding decision and infant-feeding experiences
Social implications of infant-feeding decisions	Social construction of ‘good’ mothering
	Racial discrimination, contradictions and silencing

### Information and support

#### Healthcare professionals sharing information about the BHIVA guidelines and the options available

All participants placed a high degree of value on the support and information they received from their healthcare professionals, especially their HIV clinicians. However, receiving care from multiple sources during their maternity journey (i.e. separate antenatal and postnatal services, alongside standard HIV care) was a challenge when trying to digest and consolidate information and advice. Of the postpartum women who chose to formula feed, the majority felt their HIV clinic gave them sufficient information regarding current guidelines and had supported their feeding decision. Many were offered support to access subsidised or free formula for up to one-year post-partum:

*“Yeah it was clear, they did say if you decide to breast feed we’ll support you and if you need help with formula they can refer to the charity which can provide so I was given [a] choice.”* - Sandra (32 years), two-month-old infant, formula fed

*“And then they [clinicians] were like, soon as you are living with HIV we ask mothers to bottle feed to reduce the risk of having, the baby having whatever from the breastmilk […] because they were like if you bottle feed we’ll help you with the formula milk, or we can help you breastfeed.” And I said “No it’s okay I will just bottle feed. I don’t have a problem.”* - Diablos (39 years), 11-month-old infant, formula fed

*“Oh, yes for for until the baby is a year so you can imagine what it would have been like without support, you know, if there was no support, I bet maybe most of us would stick to breastfeeding”.* - Joyce (36 years), three-week-old infant, formula fed

However, even among participants who eventually received the free formula milk provision, not receiving correct information about accessing this support initially, had required them to purchase milk themselves, which was sometimes a significant financial strain.

When care from their maternity, paediatric and HIV healthcare teams was not joined up, participants’ access to information was fragmented, which impacted health messaging. For example, Fiona described herself as a conduit between different specialist teams; her obstetric team did not have access to her HIV medical records, with consequent impact on discussions about her feeding options:

*“So*,* the first thing I wanted to talk about [with my doctor] was breastfeeding*,* so I’ve always been told in the past that breastfeeding is dangerous that you can’t breastfeed even when undetectable blah de blah de blah*,* but then because of all the stuff that I had done with*,* working with HIV I now know we can breastfeed with HIV but the research into it isn’t existent in the UK. So*,* my thing was I was writing all my questions down I was going to the maternity doctor consultant and saying right I would like to breastfeed*,* she was like you can breastfeed*,* I was like fab*,* so what do I have to do if I breastfeed? She said you need to speak to your HIV consultant*,* right okay. Spoke to the midwife as well kind of got the same response*,* [she said] “I don’t know if you can breastfeed […]*,* we don’t really know much about HIV we leave that up to the consultants to discuss that with you*,* then kind of we learn with you because we don’t see many HIV patients” and I said*,* “that’s totally understandable if you don’t see HIV patients how are you meant to know about it but then this comes into the criteria of why are you not being taught about it.”* - Fiona (32 years), eight-month-old infant, formula fed

In contrast, sometimes there was value in multiple sources of information and support, and this was especially prominent when women wished to breastfeed. It was also particularly noticeable among women who had acquired HIV in infancy (such as Fiona and Marella below), and had therefore been engaged in HIV care for several years, and were especially well-informed about UK guidelines, including how they had evolved over time. They shared how their links beyond their HIV clinic from their own experience of working in HIV advocacy enabled them to ask for extra support from other HIV clinicians and gather information from biomedical conferences:

*“[I] was going in [the clinical appointment] with lists, going right, I want to breastfeed but obviously even though I’ve been to the medical conferences [I know] we don’t have enough research on this.”* - Fiona (32 years), eight-month-old infant, formula fed

When Marella experienced “resistance” from her paediatrician regarding her plans to breastfeed, she approached another HIV clinician she knew (outside of her clinic) to speak with the paediatric team about her breastfeeding options. This resulted in her paediatrician being more supportive of her decision to breastfeed:

*“[The paediatrician] had said how reassuring it [conversation with external HIV clinician] had been and how actually what we needed to do was take it one step at a time […] and that ultimately that they needed to support my decision as opposed to working against me and to obviously keep me engaged into clinic care because obviously they didn’t want me to then disappear and start doing my own thing. But yeah, the conversation, obviously I wasn’t there as part of the conversation between them [paediatrician and external HIV clinician], but whatever was said was extremely helpful because it managed to just calm them [internal medical team] all down [previously] I felt I was continually sort of having to like reassure or manage [their] anxieties.”* - Marella (30 years), pregnant, plans to breastfeed

HIV clinicians were not only a source of medical care and information; they also referred women for additional support, such as peer support. Some women described being offered peer support, while others asked their clinicians to connect them. This desire to meet others in the same situation was particularly strong in those considering breastfeeding.

#### Women’s knowledge and perception of guidelines, transmission risk and current research

Most participants were aware that the risk of vertical HIV transmission via breastmilk (while they were virologically suppressed) was low, but were often not aware of the numerical value of risk, or overestimated the risk. Half of our participants reported that their HIV clinical team had informed them of the current UK guidance on HIV and breastfeeding, thus enabling them to make an informed decision, regardless of their infant-feeding choice. Eriife felt she had been well-informed by her healthcare team, which gave her confidence in minimising the risk of HIV transmission while breastfeeding:

*“Yeah she was very good at explaining things, what I should and shouldn’t do. My doctor was very adamant for just exclusively to breastfeeding, always, reminding me not to mix and stuff like that. Yeah […] the team have been so good that I haven’t had any, thankfully, I haven’t had bad experience of that. Probably that’s why I’ve […] been settled about breastfeeding, because any questions I had, any concerns I had, I always got them answered.”* - Eriife (33 years), 14-weeks-old infant, breastfed

Two participants were diagnosed with HIV late in pregnancy (their earliest opportunity to access HIV testing due to migration) and were therefore not virologically suppressed at the time they gave birth. Both made a fully informed decision to formula feed in accordance with BHIVA guidelines.

Half of participants reported either not being fully informed of the latest guidelines or unaware of the low risk of transmission; a minority had no recollection of a conversation with their HIV clinician about breastfeeding as an option at any point during their pregnancy. Joyce recalled how she decided to formula feed her baby following her HIV clinician’s (inaccurate) warning of the significant risk of transmission despite being virologically suppressed:

*“…they told me the risk sometimes, you know why breastfed babies, but I guess the new research that some, some patients they do breastfeed but, you know, it’s like 50/50 chances [of transmission], you know. I don’t want a situation whereby a child I was breastfeeding and they’ll have caught it, you know, so those are my fears so that was why I just stick to bottle feeding […] they told me all the risk, you know, so I have to take my choice, yeah.”* - Joyce (36 years), three-week-old infant, formula fed

While pregnant Deborah reported that she was discouraged from breastfeeding (despite meeting the BHIVA criteria):

*“they [HIV clinician] said that they encourage you not to, I said ‘no I will do it’ […] Then I breastfed that I did for two months but it because is my choice they didn’t say they, they didn’t have anything to say, they say ‘okay that is good but if something go wrong it’s your own fault because we told you’.”* - Deborah (44 years), 11-month-old infant, breastfed

Deborah also reported that she did not share when she was having difficulties with breastfeeding (detailed below in the *Social construction of ‘good’ mothering* sub-theme).

A few who had already given birth said they would have considered breastfeeding had they known it was an option:

*“I was just made to believe that [breastfeeding] was a high risk of transmission and that it was […] just a no go. It was an option if I was willing to risk that kinda thing*,* which obviously I wouldn’t so I didn’t know that there was an option to do it in a safer way*,* especially as I was undetectable way before I gave birth.”* - Amy (31 years), 12-month-old infant, formula fed

The realisation that breastfeeding could have been an option was not always met with regret, frustration or anger. Some participants stated that they would have still chosen to formula feed, even if they had known. Having successfully formula-fed older children, their desire for breastfeeding was less acute, especially where there was less cultural or peer pressure to breastfeed. On learning about the guidance now supporting breastfeeding (her youngest child now a few months old), Sinead stated she remained comforted and “reassured” by formula feeding:

*“I don’t think it would have impacted the decision having done it once the way that we did it and felt that that was safe and it was good.”* - Sinead (42 years), nine-month-old infant, formula fed

Regardless of their infant-feeding decision, and whether they had known about the most recent guidelines during pregnancy, most participants welcomed the changes:

*“[The UK guidelines are] a much needed accommodation to having a more inclusive policy that well reflects the complexities of life […] women are gonna have different desires around breastfeeding for different reasons.”* - Kay (31 years), pregnant, plans to breastfeed

One participant who had breastfed, advised against it. Her situation was unique to all other participants in that no one in her personal network (including her husband) was aware of her HIV status. She found having sole responsibility for keeping her baby safe from HIV transmission (i.e. preventing her husband from feeding their baby solids) intensely stressful:

*“I would advise people not to breastfeed really […] it’s really stressful. You might be 100% sure that [er] you’re taking your medication, [but] to me it was really stressful. […] I was living in pressure, you know, stressful. Because even at my age, I knew I was taking my medication every day, yeah? I wasn’t feeding baby another food, even the water or anything. But, it wasn’t easy. I did not have a clear mind you know, when I go for my appointment [blood tests]. That day I won’t sleep […] Even though you know everything is perfect but I had that question mark […] I used to ask myself, where is the HIV in the body?”* - Biola (39 years), eight-month-infant, breastfed

#### Making the ‘healthiest’ choice for the baby

All participants were driven by the desire to maximise the health of their babies, regardless of their ultimate feeding choice. They understood that the ‘breast is best’ messaging was complicated by the risk of HIV transmission (however small), and for most participants this resulted in choosing to formula feed, thereby prioritising having an HIV-free child:

*“I wanted to protect my baby […] I don’t want to take any risks, that’s why I went for bottle.”* - April (40 years), three-months-old, formula fed

*“I came to [the] conclusion that for the safety of the baby I will not breastfeed, I will not breastfeed, I will bottle feed.”* - Emily (41 years), five-month-old infant, formula fed

For those with an undetectable viral load and in favour of breastfeeding, the low risk of HIV transmission was offset by the health benefits, for their babies and even for themselves. Kay (who had a history of mental ill health) worried that not breastfeeding would increase her risk of postpartum depression. Others, such as Camille (below), wanted their babies to have the bonding, health and nutritional benefits from breastfeeding. As a healthcare professional herself, Camille felt she understood the data and could minimise the risk of HIV transmission while breastfeeding:

*“Because I know that obviously in terms of the nutritional value it’s better than cow’s milk, isn’t it, naturally that’s how it’s supposed to be. So, I wouldn’t have wanted anything else than to give the baby what I know nutritionally is 100% better and not only that the bond, you know, between me and the baby, you know, even just the few times I connecting to each other, you know. So it was, it was a bit emotional for me, you know, that it didn’t happen.”* - Camille (44 years), nine-month-old infant, changed to formula

### Practicalities of implementing infant-feeding guidance

#### Breastfeeding ‘safely’

While participants mostly welcomed the option to choose between formula feeding or breastfeeding, many raised concerns about how achievable it would be to breastfeed in accordance with the guidelines. A few participants recalled seeing the ‘Safer Triangle’, which provides patient facing advice on how to minimise the risk of vertical transmission through breastfeeding (please see supplementary information) [28]. Key advice includes: (i) only breastfeeding if virologically suppressed on ART; (ii) stopping breastfeeding in the event of any breast conditions or infant/maternal gastroenteritis; (iii) attending monthly routine monitoring of maternal and infant HIV viral load (via blood tests); and (iv) breastfeeding exclusively i.e. not mixing breastfeeding with either formula or solids. Sometimes, this advice was perceived as practically difficult to implement, resulting in participants opting for formula feeding:

*“[…] I thought that sounds very complicated […] I’m kind of risk averse in general, so even if theoretically there is a slight risk in breastfeeding then I would not sleep at night.”* - Sandra (32 years), two-month-old, formula fed

*“But for my doctor she just gave me the option that the, for breastmilk I can’t mix it with anything, just purely breastmilk and I have to do it for six months and during the six months I’ll be coming for check, for check-up I can’t remember if it’s monthly or weekly I don’t know, but I will be coming for check-up and it was when she said [I cannot give the baby], “Not even water.” It was that one that scared me, but I didn’t ask her, “What if the baby is having his bath and water goes in?” but I didn’t ask her but that was my big major reason I just backed out of it, breastfeeding.”* - Marcy (24 years), one-month-old, formula fed

*“Well before I got pregnant, I thought I could breastfeed but then I think around eight months in my third trimester and got to understand that breastfeeding was a lot, it was gonna be stressful for me and the baby so the best option was to formula feed and that there wasn’t enough evidence out there to show that […] I cannot pass it onto her. […] I wasn’t ready to go through that stress going to the hospital every, every month or two months. I just decided to formula feed.”* - Pauline (29 years), eight-month-old, formula fed

As a mother of twins, Tina felt that the Safer Triangle would be particularly difficult to follow successfully:

*“… the current guidelines are, well the research rather is based on full term babies and the safety is based on purely breastfeeding for six months and not combination feeding and our twins were born at 31 weeks so very early and there’s two of them. So the fact that the safety data isn’t there for premature babies yet and the fact there’s two of them I just felt that solely breastfeeding would be very difficult, I know some people manage but I think, you know, the number that manage to exclusively breast feed multiple babies is, is much lower than a singleton and the safety data isn’t there for premature babies. So I spoke to my consultant and chatted it through with her and we decided that, well I decided ultimately that, you know, safety first sort of thing so decided not to. But luckily where I am the NICU, because they were in intensive care for eight weeks well the special care baby unit for eight weeks they have access to a breast milk bank so they had donor breastmilk for the first sort of three weeks which was great and then went onto the premature baby formula.”* - Tina (36 years), 7.5-month-old twins, formula fed

Women’s experience of breastfeeding was vastly improved when they received more personalised information and support. For example, Gracelove recalled her medical team providing practical advice on how to manage breastfeeding in the event of different health issues, including how she could resume breastfeeding after a brief pause:

*“I was also advised to express and then freeze, freeze it just in case I became unwell and I didn’t want to stop [breastfeeding] then I could use the frozen once I became well enough to be breastfeeding. But then during that time [that I am unwell] I would have to express and pour away [the breastmilk]…”* - Gracelove (38 years), sixth-month-old infant, breastfed for four months

In the absence of this kind of additional personalised and supportive care, national guidance was regarded as impractical and seen to be placing an unfair burden on women:

*“In my opinion, the current triangle policy is not human-centred […] is not an ideal policy response. […] The guidelines as they currently stand do not really empower HIV positive women, they guilt and suffocate them.”* - Kay (31 years), pregnant, plans to breastfeed

#### Congruence between infant-feeding decision and infant-feeding experiences

Regardless of mode of feeding, postpartum participants felt increased confidence in their decisions when seeing their babies grow healthy and HIV free. For those who had opted to formula feed, there were initial practical challenges related to preparing feeds, however they soon adjusted to this routine, sometimes supported by partners:

*“Now it’s okay, I’m used to it, but to start with it was a bit challenging and, you know, when you bottle feed you have to prepare the bottle and clean them, clean them out, making the milk, you have to get up in the middle of the night to go and make milk. But it was really hard to start with but things are getting better.”* - April (40 years), three-months-old, formula fed

Likewise, our (smaller) set of participants who had successfully breastfed their babies (with no reported HIV transmissions), reported feeling validated in their decision to breastfeed when their babies grew well and they themselves enjoyed breastfeeding:

*“Oh, I loved breastfeeding, I loved it. I loved every second of it. I did not find it difficult; I mean, at times it was demanding but […] I just loved every second of it to be honest, you know, I did not find it challenging. I had enough breast milk supply at all times, you know, I loved it and my son loved it too.”* - Puleng (29 years), 11-month-old infant, breastfed for eight months

Generally, those who had breastfed felt that breastfeeding was easier as it did not require the same preparation as formula feeding, while those who formula fed felt the opposite.

#### Incongruence between infant-feeding decision and infant-feeding experiences

A few of our participants had planned to breastfeed but were unable to breastfeed or had to stop breastfeeding earlier than planned. Stephanie stopped breastfeeding after a few days because of cracked nipples (as per national guidelines):

*“[M]y partner [and I] decided straight away to [breastfeed] that first month, maximum one month but actually we could only for a few days because after my breast started to be painful and it [nipples] started to be cut.”* - Stephanie (40 years), six-months-old, changed to formula

Switching to formula had little negative emotional impact on Stephanie as she had planned to breastfeed for a short amount of time anyway. In contrast, two participants described below (who had planned to breastfeed) had been advised by maternity healthcare professionals to start formula-feeding following difficulties establishing breastmilk supply immediately post-partum, which was upsetting for them. A lack of clarity in national guidelines then led these healthcare professionals to advise both women not to re-start breastfeeding due to a perception that this would fall under the category of ‘mixed feeding’ (which is not advised in the context of HIV):

*“The first day I breastfed, it was good like I said I was really happy doing it and I was just caught unaware. The decision [from the maternity staff] was just instant [they said], “he needs this amount of fluid” you know, and my milk is not producing it like give me a chance, it didn’t make no difference though, for you to know that it wasn’t necessary because despite the formula milk it still didn’t get rid of the jaundice […]until they did further treatment and by that time the breast milk was gushing out, so the support was really poor.”* - Camille (44 years), nine-month-old infant, changed to formula

Feeling desperate, Camille tried accessing breastfeeding support, without success as it was outside office hours. Another participant described how her baby was given formula soon after birth, when her partner was present in the room. Since he did not (yet) know about her HIV status, and she was “exhausted”, she did not protest. However, later she was advised not to establish breastfeeding due to a perceived risk of mixed feeding:

*“I had a very long tiring labour so, by the time it was done, I was so exhausted. […] So the nurse who was looking after me that day. I think she asked something about giving him some [formula] milk, right? And so, me, I thought she knew [my HIV status], I thought she knew what was going on…, and my partner didn’t know [my status] at that time. So, […] I just didn’t want to be like “Oh no, no he can’t have this milk,” or something like that. So, I don’t know, in my mind, when she gave him the milk, I thought maybe it was okay as a one off. […] And the next thing they’re coming and telling me like “Oh no, because we’ve already given him this [formula] milk you can’t breastfeed anymore.” And they were quick enough to give me the pill to dry up my [breast] milk, very, very quick.”* - Nozipho (30 years), 11-month-old infant, formula fed

Rachel had explored her infant-feeding options before her first pregnancy because breastfeeding was “something personal to me that I’ve wanted to do […] because I’m an African.” However, she had a complicated birth and her baby spent an extended period of time in hospital. Below Rachel describes her experience of pumping milk for the maternity ward to give to her baby and her interaction with the medical staff when they told her it was not advisable:

*“So I remember there was one time she called me, she was like, [um] “Are you, [um] what’s the plan now and how many breast milk are you pumping.” […] I’m like, [um] “I’m not pumping that much.” And she said, “Because you’re not pumping that much, we can’t advise you to give it to the baby because she has to be exclusively on breast milk but you’re not producing much.” […]I used to express, but me taking it to the hospital for her, they were stopping me, you know, to give it to her. You know, like, “We know this is your status and we don’t want to put baby in the risk.” […] So all the milk that I had been taking, express for her, they just put it in the sink.”* - Rachel (30 years), seven-month-old, formula fed

In total five women in our sample had planned to breastfeed but either could not at all or stopped significantly earlier than they had originally planned: for Stephanie and Christine this was due to cracked nipples; meanwhile Camille, Rachel and Nozipho were advised that they did not have enough milk supply to breastfeed exclusively, so should not start/continue to avoid mix-feeding;

In contrast to the above, Holly (still pregnant) had planned to formula feed, but at the time of her interview, had changed her mind (and now wished to breastfeed) when she saw how breastfed babies in her family “just chunk up real quick and it does make them look really healthy and a bit more robust.”

As these examples illustrate, participants’ associated barriers and ease with both feeding options (either due to perception, advice from others, HIV secrecy or previous experiences) impacted their infant-feeding decisions, at least in part.

### Social implications of infant-feeding decisions

#### Social construction of ‘good’ mothering

International HIV guidelines recommend that women living with HIV breastfeed, and ‘breast is best’ remains a widespread and powerful public health message in the general UK population as well as internationally. This led to our participants feeling conflicted, challenging their own internal notion of what ‘good’ mothering is. This was particularly, but not exclusively, felt by women who were themselves (or partners to those) of racially minoritised backgrounds, because of a stronger cultural norm that ‘good’ mothers breastfeed:

*“It’s a very natural thing to just want to [breast] feed the baby and obviously in the African community you need to breastfeed so it was preparing myself for the questions* [from my in-laws] *of “why aren’t you breastfeeding”*,* “why are you formula feeding”*,* and having a story ready that was my big like thing of having a story to just shut everybody up and leave me alone kind of thing because obviously we’re not gonna be talking about the diagnosis with the family and stuff like that. I remember the build up to having the baby I was panicking about those questions*,* and they do like to question you a lot yeah.”* - Amy (31 years), partner to Black Southern African, 12-month-old infant, formula fed

Even when supported to breastfeed, some participants found that BHIVA guidance (for example, to stop breastfeeding by six months) did not match their own expectations, and experiences of breastfeeding among family and peers. This left some conflicted; having to choose between adhering to medical advice and exercising their autonomy to feed their baby in a way they thought was best. A few breastfeeding participants reported non-adherence to the BHIVA and Safer Triangle breastfeeding guidance in various ways. Deborah breastfed despite having cracked nipples; Puleng breastfed beyond the recommended six months and occasionally mixed-fed (with solids) her eight-month-old baby. Maria breastfed to 15 months^1^. While Deborah and Puleng did not share their feeding practice with their healthcare professionals for fear of being told to stop, Maria had been supported by her HIV clinician to breastfeed beyond a year. However, when she moved to a different HIV clinic, she was challenged by her new HIV clinician:

*“So the [new] doctor called me and said oh, you know, ‘oh we’re in really deep water because, you know, I thought you’d stopped feeding and, you know, you shouldn’t be still, you shouldn’t still be doing it, you know, he’s 15 months now and, you know’, I think he was saying, I don’t know what he, like he really panicked me because he was like, ‘you know, the guidelines are that he should stop after six months, and you know, you’ve already done it for long enough now and, you know, you need, basically’ saying to me you need to stop as soon as possible. So I kind of panicked I was like, you know, what’s going on and, you know, am I putting my son in danger and what, what is it, what is the reason why he’s kind of telling me to stop this, but, but I think I didn’t really question it.”* - Maria (37 years), 16-month-old infant, breastfed for 15 months

Ultimately, all these participants felt happy with their decision as their babies were HIV free. These cases illustrate the ongoing nature of infant-feeding decision-making which extends from pregnancy through to potentially many months postpartum. Moreover, while the quotes above spoke of cultural pressures to breastfeed, Amina and Nozipho (South Asian and Black African respectively, and both British born) reported less cultural pressure to breastfeed, suggestive of shifting expectations for their (younger) generation.

#### Racial discrimination, contradictions and silencing

Racially minoritised participants in particular sometimes described conversations with some healthcare professionals about their infant-feeding decisions as fraught. Their experiences of these interactions were marred by HIV stigma, racism and anti-immigrant rhetoric.

Lana’s professional experience as a clinical research nurse in West Africa meant she was well informed about current HIV and breastfeeding data. She raised the potential limitations of existing research with her HIV clinician, wanting to engage in a deeper conversation about the potential risks of breastfeeding and validity of the data informing the UK guidelines. However, she felt that her clinician did not acknowledge her expertise, and potentially racially stereotyped her:

*“…the few questions I’ve asked them and clearly no-one, no-one at all has answers because I even asked one of the doctors […] They didn’t give me [an] answer, […], I think like I said maybe because I’m a Black person they don’t understand that I have research understanding […] Most policies are informed by research and research can be outdated […] Maybe something may have changed so when I ask them questions, they treat it like, “Oh she doesn’t have enough knowledge, or she may not understand.”* - Lana (39 years), pregnant, undecided

Puleng (Black African), mentioned earlier, recalled feeling silenced and discriminated against during her labour; while this experience was not specifically regarding infant-feeding, she was still traumatised by it and it had eroded her trust in healthcare. However, it is also important to note that experiences of discrimination within maternity and HIV services were not universal, as illustrated by Gracelove (Black African) above (who breastfed) and Rachel (Black African):

*“Really no one has ever treated me differently. I can’t lie about that. No one, no healthcare worker around here has ever treated me differently […] you have your doctor to look after you, you have a special doctor for that and a specialists for women that are living with it that are pregnant.”* - Rachel (30 years), five-month-old baby, formula fed

None of the above attended the same NHS Trust as one another. Moreover, the minority of participants who did report experiences and perceptions of racial and anti-immigrant discrimination stated that the medical staff were from various ethnic backgrounds.

## Discussion

Despite the most recent UK guidance stating that breastfeeding can be supported in certain situations, our research suggest the support is inconsistently applied within clinical settings, and many mothers living with HIV have only partial (both meanings of the word) knowledge about their options regarding infant-feeding. Participants had varied levels of knowledge about current infant-feeding guidance, and were often not aware that the risk of vertical transmission was very low in the context of maternal virological suppression. This lack of knowledge constrains women’s choices, preventing truly informed decision-making. Healthcare professionals’ lack of knowledge of and/or confidence in updated guidelines leads to inconsistent management of infant-feeding in this group, with some women receiving appropriate advice and support, and others being given incorrect information. The Lancet’s Breastfeeding Series calls for a greater general recognition of the impact on global promotion of commercial formula milk on breastfeeding rates and attitudes toward formula feeding among families and healthcare professionals [37]. Specifically, current UK HIV and infant-feeding guidelines encourage formula feeding, within a wider national context where breastfeeding initiation and continuation rates are some of the lowest in the world [38, 39], which may make advocating for breastfeeding harder in this context, despite mothers with HIV being more likely to belong to minoritised communities where breastfeeding is the norm. Some participants reported being actively dissuaded from breastfeeding by their healthcare professionals, which implies a continuing and exclusive focus on averting the risk of vertical transmission above all other considerations, such as the potential health, social and emotional benefits of breastfeeding to both mother and baby. Additionally, HIV-related stigma and racial discrimination when accessing healthcare, further disempowered several of our participants, impacting interactions with healthcare professionals and leading to medical mistrust.

While the evolution of the guidelines were generally welcomed among our participants, they do present more complex infant-feeding decisions. Additional choice and freedoms, especially when not matched with appropriate viable practical advice and support to implement them, create unique tensions and complexities that paradoxically, do not exist under stricter, more unilateral infant-feeding policies. Participants reported that this friction was compounded when healthcare professionals were not transparent or supportive of their options (namely choosing to breastfeed). It is ironic that guidelines that promote increased autonomy and agency have potentially created additional internal and external frictions for pregnant women and mothers living with HIV as they negotiate and manage risk. Whereas, previously all women were advised to formula feed, the introduction of choice has allowed infant-feeding decisions to now be laden with moral value among women with HIV and their healthcare providers; depending on the perspective, both breast and formula feeding can be viewed as transgressive [32].

To our knowledge, this is the first qualitative study that captures infant-feeding considerations and decision-making among women living with HIV in the UK since the BHIVA guidelines changed in 2018 (please note that at the time of writing, the 2024 guidelines were being drafted for public consultation). Our findings reinforce that pregnant women and mothers living with HIV continue to face complex medical, emotional and psychological challenges during the perinatal period [40]. Our data supports existing literature which shows breastfeeding avoidance continues to cause emotional distress, particularly for African migrants [29, 41]. Resonating with our findings, a metasynthesis found that women with HIV (in high-income settings) who did not breastfeed risked increased internalised HIV stigma, impeded sense of worth as a mother, felt disconnected from their culture and faced greater surveillance from their peers [42, 43]. In a US-based breastfeeding avoidance study, one-in-five women with HIV experienced post-partum depression one month after delivery and a significant proportion of participants associated breastfeeding avoidance with feelings of “sadness ” or a “lack of empowerment” [44]. As we found in our study, Black migrant women in the UK struggle while formula feeding their babies due to the cultural expectations to breastfeed, the risk of signalling their HIV status when formula feeding, and the specific and situated challenges they face being positioned at the intersections of their gender, race, ethnicity and migrant status [29, 41, 43].

While HIV stigma and poor knowledge among non-HIV specialist settings is widely acknowledged [45], our data provides novel insights into the stigma and poor knowledge and communication among HIV clinicians, as perceived by their patients. This resonates with data from South Africa that showed an absence of accurate and up-to-date knowledge of latest infant-feeding policies and data among clinicians [46, 47]. In our study, even where our participants reported good communication and information support from their HIV specialist clinicians, maternity care services being separate and siloed from HIV care resulted in some healthcare interactions requiring women to educate the healthcare professionals and advocate for themselves, which they found intensely stressful. Our data corresponds with other literature that explores power relations within the context of pregnancy and congenital health conditions [48–50] resulting in risk ownership and risk management being held by some healthcare professionals exclusively, especially when pregnant women and mothers had limited awareness of the national guidelines and data influencing them.

### Implications for clinical practice and areas for future research

Our findings highlight the need for early, non-judgemental and evidence-based infant-feeding conversations between well-informed clinicians and parents, that continue throughout the maternity journey. This recommendation is relevant to all parents; having older children is no guarantee of women’s confidence in their infant-feeding decision-making or their awareness of the latest HIV guidance. To ensure women’s autonomy to make an informed infant-feeding decision, supportive non-judgemental guidelines must be coupled with practical and up-to-date advice. For example, information regarding where and how to access free formula and support services that may help with immediate breastfeeding advice. Pharmaceutical innovation, including greater understanding of long acting injectable ART, may affect attitudes towards and guidance regarding breastfeeding while living with HIV. Healthcare professionals should discuss the opportunities and challenges of breastfeeding beyond HIV transmission risk, and ensure that prospective mothers are aware of the health and wellbeing benefits to them and their baby [16, 18–21, 51]. Peer support may provide additional support and access to resources between clinical appointments; peer support has been repeatedly shown to improve the health and experience of people with HIV generally [52] as well as mothers specifically [53].

It is important that healthcare professionals, whether operating as a multidisciplinary team or separate specialisms, share consistent up-to-date information about HIV and infant-feeding options. While HIV specialists should take the lead within these conversations, women are likely to discuss their new pregnancy and subsequent breastfeeding challenges with their primary health carers. In fact, their infant may receive care from neonatologists or other paediatric sub-specialties, while maternity staff will be the healthcare professionals available when the guidance is first implemented in practice. As such, all these medical specialties should have knowledge and confidence in the latest BHIVA guidance.

Our findings raise concerns about healthcare professionals’ knowledge and confidence to share the latest BHIVA guidance. Further research should explore how medical staff’s approach to sharing the latest UK HIV and infant-feeding guidance impacts their patient interaction and decision making. There is insufficient research on this in the UK, however a study (cited earlier) on the experiences of South African based frontline workers showed a disconnect between policy and practice within health settings [47]. Pregnancy-related risk may be perceived differently by pregnant women and their clinicians [54, 55] and therefore clinicians withholding information, even when well-intended, may result in mistrust and covert infant-feeding practices. A transparent approach, where women are counselled based on up-to-date guidelines and offered a truly informed choice, is required for BHIVA to maximise its potential for standardised care across the nation and reach its own aspiration of fully informed, empowered infant-feeding decisions. BHIVA was immediately responsive to the data we presented at the 2023 BHIVA Conference, clarifying its position on mixed-feeding guidance [56]. Understanding how experiences of breastfeeding change within this new context will provide valuable insights to how clinicians and parents perceive the practicalities of breastfeeding (specifically, whether the guidance will continue to be viewed as impractical by mothers and birthing parents).

## Strengths and limitations

This study includes a large sample of mothers with HIV, across England and Scotland, however, we do not have accounts from anyone based in Northern Ireland or Wales. Our sample was diverse in terms of age, ethnicity and range of infant-feeding experiences such that we were able to present a comprehensive picture of infant-feeding decision-making in the context of HIV in the UK. Aside from academic publications, the NOURISH-UK research is also published as a support and information website at: https://hexi.ox.ac.uk/Feeding-a-baby-while-living-with-HIV/overview. The NOURISH-UK study was timely and needed; presentation of our early findings at an HIV conference informed the release of an urgent position statement clarifying the meaning of mixed-feeding [56], and are informing the BHIVA guideline update due in 2024.

Despite the diverse clinical experiences shared by our participants, healthcare professionals were not included in this study, so we relied on accounts from recipients of care. It may have been of benefit that both researchers were from a non-clinical background, especially for participants who reported negative experiences within the healthcare system. Elsewhere (manuscript currently under review) we reflect on how having racially minoritised researchers, and stakeholder engagement and co-production with Black women living with HIV who were part of the study team and wider advisory panel, deepened our reflexive practice and generated novel insights [36]. Finally, data were collected during the COVID-19 pandemic, some during lockdown, which affected participants’ contact with their medical teams and their experiences. The pandemic also impacted our interview methods: participants were offered a choice of telephone or online video interview (with a further offer to courier devices to their place of choice and provide data) in order to widen accessibility. Qualitative researchers are increasingly using remote data collection methods; it provides participants with options and visual cues can still be noted (via video data collection) however data quality may be affected when participants’ cameras are off [57]. One scoping review found that ‘online methods may increase the likelihood of obtaining the desired sample, but responses are shorter, less contextual information is obtained, and relational satisfaction and consensus development are lower’ (Davies et al., 2020) [58] Generally, we believe that conducting these interviews remotely allowed for greater access, especially among participants who were close to their due date or fewer than six weeks postpartum.

## Conclusion

Regardless of their feeding decision, all participants focused on what they deemed to be the *best* choice for their baby. The 2018 changes in the UK infant-feeding guidelines are not reflected in the experiences of the women interviewed in this study. There is a significant informational need among women with HIV around their infant-feeding options and understanding the risk of HIV transmission via breastmilk. Moreover, women deserve to be informed of the risk and benefits of breastfeeding and formula feeding to ensure they are able to make an informed decision about their infant-feeding options. Our study suggests that some healthcare professionals are either unaware of updated guidelines or are unwilling to implement them in practice. Women should be entrusted with the latest clinical guidance and practical support and advice in order to empower them to make the best choice for their families. Specific training regarding BHIVA’s infant-feeding guidelines for all healthcare professionals who support pregnant women and new mothers with HIV will help to empower women to make fully informed infant-feeding decisions. There also needs to be greater cohesion between HIV specialists, maternity specialists, paediatricians and other healthcare professionals to avoid conflicting messaging and to ensure greater cohesion within clinical care.

### Electronic supplementary material

Below is the link to the electronic supplementary material.

Supplementary Material 1

## Data Availability

The hexi.ox.ac.uk *Feeding a baby while living with HIV* web resource may aid conversations between healthcare professionals, women and their partners (https://hexi.ox.ac.uk/Feeding-a-baby-while-living-with-HIV/overview) includes additional data from this study. All participants and/or their legal guardian(s) provided informed consent for publication of any identifying information/images contained on the website.

## Abbreviations

ART: Antiretroviral therapy
BHIVA: British HIV Association
HCP: Healthcare professionals
HIV: Human immunodeficiency virus
MDT: Multidisciplinary team
MS Teams: Microsoft Teams
NHS: National Health Service
NIHR RfPB: National Institute for Health and Care Research Research for Patient Benefit
PIC: Participant Identification Centres
PIS: Participant information sheet
PPI: Public and patient involvement
U = U: Undetectable equals Untransmittable
UK: United Kingdom
US: United States
UNICEF: United Nations Children’s Fund
WHO: World Health Organization

## Footnotes

1 Participant was included in the study, despite being > 12 months postpartum, due to her unique experiences.
