# Supplementary material for: How women living with HIV in the UK manage infant-feeding decisions and vertical transmission risk – a qualitative study
Source: BMC Public Health. 2024 Aug 6;24:2130. doi: 10.1186/s12889-024-19581-9 (PMC11302277; doi:10.1186/s12889-024-19581-9)
Supplement: Supplementary file 1 — Supplementary Material 1 [file 12889_2024_19581_MOESM1_ESM.pdf]

HIV and feeding your newborn baby

The safest way for a person living with HIV in the UK to feed their baby is to bottle feed using formula milk.

If you are on HIV treatment with an undetectable viral load and choose to breast/chestfeed, we can help you make it as safe as possible for your baby. However, it will not be as safe as using formula; there is no risk at all using formula. Until we know more about the safety of breast/chestfeeding on HIV treatment, this guidance will give your baby their best chance of remaining HIV free while breast/chestfeeding. Always protect your baby using 'The Safer Triangle':

No virus

If the HIV virus is detectable in your blood, there will be HIV in your milk, and HIV will enter your baby's body during feeding. You should only breast/chestfeed if you are taking treatment and your HIV is undetectable

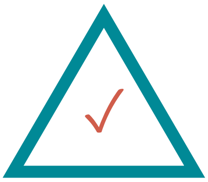

Healthy tummies

Diarrhoea and vomiting show that a tummy is irritated. If your baby's tummy is irritated, it may be more likely that HIV will cross into their blood stream. If your tummy is irritated, you may not absorb your anti-HIV medication properly. Only breast/chestfeed if both of you have a 'healthy tummy'

Healthy breasts/chest

There may be HIV in your milk if your nipples are cracked or bleeding, or if you have thrush or mastitis. Only breast/chestfeed if your breasts/chest and nipples are healthy

The Safer Triangle means:

No virus **+** *healthy breasts/chest* **+** *healthy tummies*

Only breast/chestfeed if your HIV is undetectable

AND

both you and your baby are free from tummy problems

AND

your breasts/chest and nipples are healthy with no signs of infection

If HIV virus becomes detectable in your blood: stop breast/chestfeeding and start using formula milk. Do not use milk you have expressed and stored. Feed your baby using formula only. Your baby may need to take anti-HIV medication as post exposure prophylaxis. Contact your clinic team to discuss this urgently.

If your baby is unwell with diarrhoea and/or vomiting: feed your baby with formula milk only while your baby is unwell. As it can be difficult to know when the baby's gut lining has fully recovered, we do not advise restarting breast/chestfeeding, but continuing formula milk. Contact your HIV team for advice on what to do.

If you have diarrhoea or vomiting, or a breast/chest injury or infection: stop breast/chestfeeding and feed your baby with formula milk OR use milk that you expressed more than 2 days (48 hours) before your tummy or breast problem began. You may return to breast/chestfeeding 2 days after your breast/chest has healed. If you had tummy problems, you must contact your clinic team before breast/chestfeeding again.

How to help protect your baby from HIV while breast/chestfeeding

How to tell if breast/chestfeeding is going well

Sometimes it is hard to get breast/chestfeeding going, and not all parents find it easy at the start, especially learning how to make sure the baby latches on well to the nipple. At first it can also be hard to know whether your baby is getting enough milk. You can be reassured they are, if your baby has plenty of wet and dirty nappies. Don't be afraid to ask for support from your midwife, or a lactation consultant.

Taking your anti-HIV medication

Taking your anti-HIV medication at the right time every day ensures the virus is asleep in your blood, which protects both you and your baby. Taking your medication as perfectly as possible is just another part of the love that you are already giving to your child.

Short and sweet

The shorter the time you breast/chestfeed your baby, the lower the risk your baby will have of getting HIV. In a study of women breastfeeding on anti-HIV treatment (the PROMISE study; see reference below), babies breastfed for 12 months had double the chance of getting HIV compared to those who breastfed for 6 months.

No solids before 6 months of age while breast/chestfeeding

Introducing your baby to solid foods (sometimes called weaning, complementary feeding or mixed feeding) should start when your baby is around 6 months old. If your baby is less than 6 months old, they should receive only breast/chest or formula milk and NO solids.

Giving breast/chest milk with other solid foods may irritate the young baby's tummy and increases the risk of HIV passing to the baby. Ideally, before starting to wean your baby, you should transition from breast/chestfeeding to giving your baby formula milk only.

When babies are 6 months old they are ready to start being weaned, and can gradually have simple weaning foods along with their formula milk. Using only formula milk while weaning means your baby will get the vitamins and nutrients they need to grow without any risk of HIV; formula milk is still the safest way to feed your baby in the UK.

For more weaning advice, please see the NHS website: www.nhs.uk/conditions/baby/weaning-and-feeding/babys-first-solid-foods/

Be prepared, in case feeding does not go to plan

Breast/chestfeeding does not always go to plan for any parent. Although exclusive breast/chestfeeding is ideal, if you don't have enough milk, it is alright at any age to give your baby formula milk as well as breast/chest milk, if they need a top up.

Although research has not shown that giving breast/chest milk in combination with formula milk increases the risk of babies getting HIV, this is only recommended in certain situations. It is important to manage these situations with extra planning; the advice for someone breast/chestfeeding with HIV might be different from advice for others. We encourage you to tell your community midwife about your HIV to make sure they give you the right advice for you and your baby. If you are uncertain about something, don't hesitate to ask your specialist midwife, specialist children's nurse or HIV doctor; make sure you have a number to call.

Reference: Prevention of HIV-1 transmission through breastfeeding: efficacy and safety of maternal antiretroviral therapy versus infant nevirapine prophylaxis for duration of breastfeeding in HIV-1-infected women with high CD4 cell count (IMPAACT PROMISE): a randomized, open label, clinical trial. J Acquir Immune Defic Syndr 2018; 77: 383–392.

Help with infant feeding problems for people with HIV

This section lists some of the problems that may occur while you are breast/chestfeeding. Get help early, if you can. If you cannot reach a healthcare professional who understands HIV and breast/chestfeeding, use The Safer Triangle and ask your community midwife or GP for advice.

Mastitis

When milk stays in the breast/chest for longer than usual, or the whole breast is not being fully emptied of milk, you can get a blocked milk duct. This can become inflamed and/or infected, and is called mastitis. Mastitis is very common. Speak to your community midwife about how to prevent and treat a blocked duct so that you do not get mastitis.

Symptoms of mastitis

- A red, swollen area on your breast/chest that may feel hot and painful to touch.
- A lump or area of hardness on your breast/chest.
- A burning pain in your breast/chest that may be continuous or may only occur when you are feeding.
- Nipple discharge, which may be white or contain streaks of blood.
- You may also feel achy, have a high temperature and/or chills and be very tired.
- Mastitis causes the amount of HIV in milk to increase if not on treatment.

Mastitis can develop quickly. See your GP or go to A&E if you have symptoms of mastitis to avoid a breast/chest abscess forming.

If you develop mastitis

- **Do not breast/chestfeed your baby if you have mastitis**, the safest thing is to stop breast/chestfeeding and change to formula milk.
- Express and discard your milk regularly from both breasts/sides of the chest.
- Discard any milk expressed within the 2 days before the breast/chest became sore.
- Rest and drink lots of fluids, and avoid tight clothes or bras.
- Warm baths and directing a hot shower onto the affected breast/chest can help.
- You may return to breast/chestfeeding 2 days after your mastitis has healed.

Cracked or bleeding nipples

Sore and injured nipples are usually caused by the baby not latching onto the nipple well. Early help can prevent sore nipples becoming cracked or bleeding. Ask your community midwife or health visitor for help with this. Irritated and broken skin can allow your blood to get into your breast/chest milk. This could increase the chance that your baby may get HIV.

- **Do not feed your baby from the sore breast/side of the chest** while the nipple is cracked.
- Hand express or pump milk from the sore breast/side of the chest and discard this milk.
- Do not feed you baby from the sore breast/side of the chest until it is healed and has been pain free with no bleeding for at least 2 days.
- Breast/chestfeed your baby from the other breast/side of the chest.
- If both nipples are cracked and sore – even if there is no blood – then do not breast/chestfeed.
- Use your supply of stored expressed milk instead or feed your baby using formula milk.
- You may return to breast/chestfeeding 2 days after your nipples have completely healed.

Diarrhoea and vomiting in the breast/chestfeeding parent

You may not absorb your HIV medicine well if you have diarrhoea or are vomiting. This may cause a temporary increase in the amount of HIV in your blood and breast/chest milk.

- **Do not breast/chestfeed your baby if you have diarrhoea or are vomiting** because you may not have absorbed enough of your HIV medicine.
- Use your supply of stored expressed breast/chest milk or formula milk instead.
- Express your milk and discard it until at least 2 days after you last had diarrhoea or vomited.
- Tell your clinic team, as they may want to check that the virus in your blood is still undetectable. The team may ask you not to breast/chestfeed your baby, and to discard any expressed breast/chest milk, until they have been able to check the amount of virus in your blood.

Diarrhoea and vomiting in the baby

If your baby is sick with diarrhoea and/or vomiting (gastroenteritis), it is safer to feed them with formula milk and not breast/chest milk. Diarrhoea and vomiting are signs that your baby's tummy and intestines are irritated. This will make it more likely that any HIV in your breast/chest milk can enter into your baby's blood and cause infection. After a bout of diarrhoea and/or vomiting, it can take some time for the baby's tummy and intestines to fully get back to normal.
Start formula feeding and continue; do not return to breast/chest milk. Contact your HIV clinic team for advice.

If HIV becomes detectable in your blood

- If your HIV viral load becomes detectable in your blood, **stop breast/chestfeeding and start feeding your baby with formula milk**.
- Your baby may need to take anti-HIV medication as post exposure prophylaxis. Contact your clinic team to discuss this urgently.

Finally...

We are learning more all the time about how to keep parents with HIV and their babies healthy. You may have a question for which we do not yet have a definite answer. If this happens we will use our experience to guide you. We will tell you when we know about new scientific evidence. If you have a question and cannot reach us, use The Safer Triangle.

Contact details

Mentor Mothers at Positively UK http://positivelyuk.org/pregnancy/
Helen Rogers: telephone number, 020 7713 0444; Email address, hrogers@positivelyuk.org

For other organisations that can provide basic breast/chestfeeding advice, please see the NHS website 'Breastfeeding Help and Support' (https://www.nhs.uk/conditions/baby/breastfeeding-and-bottle-feeding/breastfeeding/help-and-support/) for a list of websites and helplines.

Helplines

National Breastfeeding Helpline: 0300 100 0212
Association of Breastfeeding Mothers: 0300 330 5453
La Leche League: 0345 120 2918
National Childbirth Trust (NCT): 0300 330 0700

Authors

Yvonne Gilleece, Laura Byrne, Alasdair Bamford, Nell Freeman-Romilly, Hermione Lyall, Paula Seery, Shema Tariq
January 2023
